# Supplementary material for: A Global Screen for Assembly State Changes of the Mitotic Proteome by SEC-SWATH-MS
Source: Cell Syst. 2020 Feb 26;10(2):133–155.e6. doi: 10.1016/j.cels.2020.01.001 (PMC7042714; doi:10.1016/j.cels.2020.01.001)

Q6PIU2 | NCEH1_HUMAN | NCEH1 AADACL1 KIAA1363
Monomer MW [kDa]: 45.808 Monomer expected elution fraction: 47

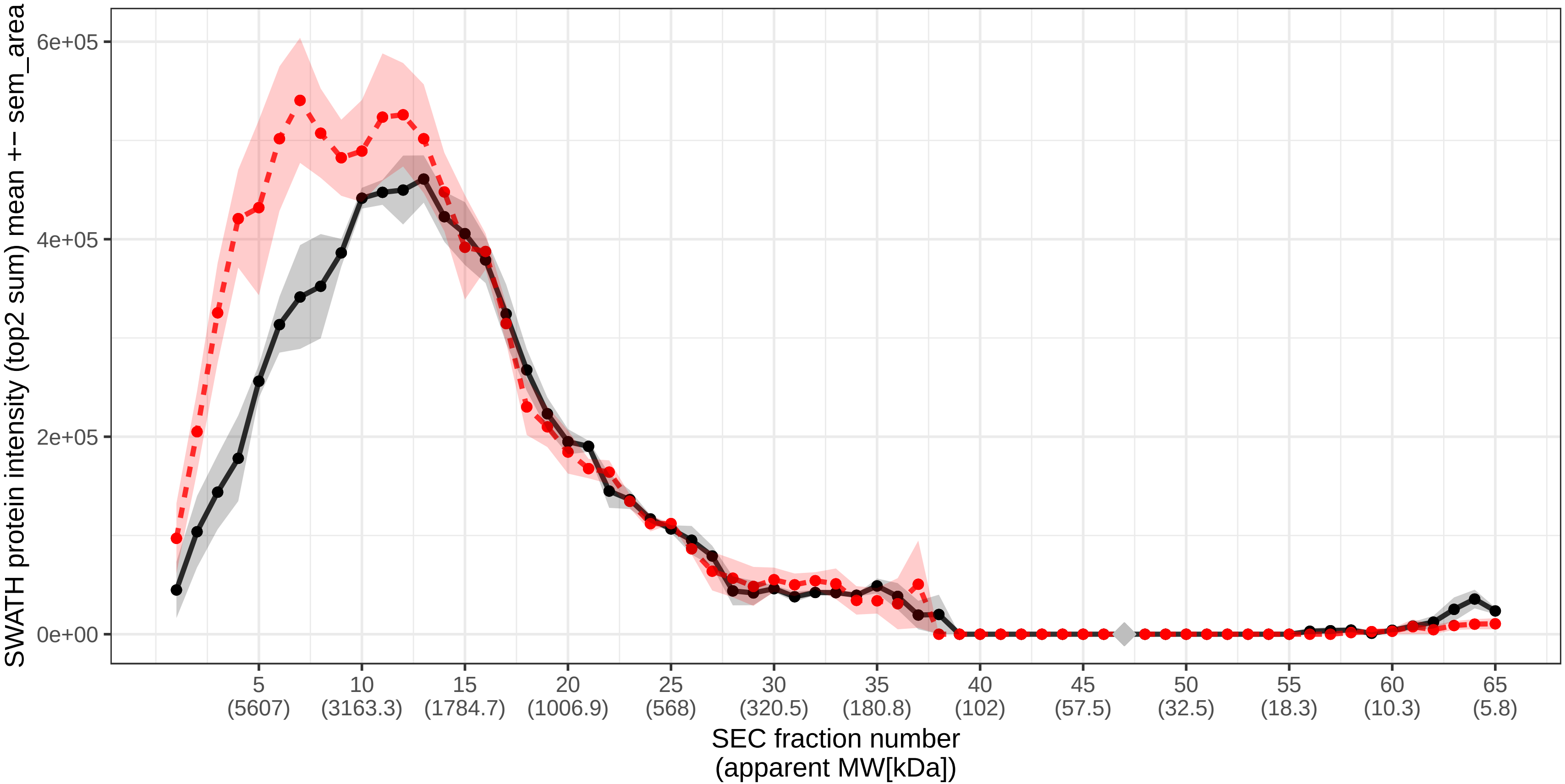

Supplement: Data S1. SEC-SWATH-MS Protein Chromatograms, Related to Figure 1 [file mmc6.zip › SECchrom_Q6PIU2_NCEH1_HUMAN_NCEH1_AADACL1_KIAA1363.pdf]
